# Emerging therapeutic frontiers in hypertension management

**DOI:** 10.3389/fcvm.2025.1550181

**Published:** 2025-05-16

**Authors:** Diana-Ligia Pena, Justin Aurelian, Mihai Grigore, Andreea-Simona Hodorogea, Emma Weiss, Elisabeta Bădilă, Adriana-Mihaela Ilieșiu, Ana-Maria Balahura

**Affiliations:** ^1^ Department of Cardiology, ^„^Prof. Dr. Theodor Burghele” Clinical Hospital, Bucharest, Romania; ^2^ Department of Urology, ^„^Prof. Dr. Theodor Burghele” Clinical Hospital, Carol Davila University of Medicine and Pharmacy, Bucharest, Romania; ^3^ Department of Cardiology, ^„^Prof. Dr. Theodor Burghele” Clinical Hospital, Carol Davila University of Medicine and Pharmacy, Bucharest, Romania; ^4^ Department of Cardiology, Colentina Hospital, Carol Davila University of Medicine and Pharmacy, Bucharest, Romania

**Keywords:** clinical trials, hypertension, novel antihypertensive agents, pharmacotherapy, resistant hypertension

## Abstract

Resistant hypertension, characterized by persistently elevated blood pressure despite adherence to multiple antihypertensive therapies, remains a significant global health challenge. This condition is associated with increased cardiovascular morbidity and mortality, highlighting the urgent need for more effective treatment strategies. While current management focuses on lifestyle modification and pharmacotherapy, many patients do not achieve target blood pressure levels. Recent advances have led to the development of emerging therapies targeting novel mechanisms, including gene silencing, receptor modulation, enzyme inhibition, and immunomodulation. This descriptive review examines the efficacy, safety, and clinical trial progress of these innovative therapeutic approaches, offering new hope for improved blood pressure control in resistant hypertension.

## 1 Introduction

**Figure 1:**
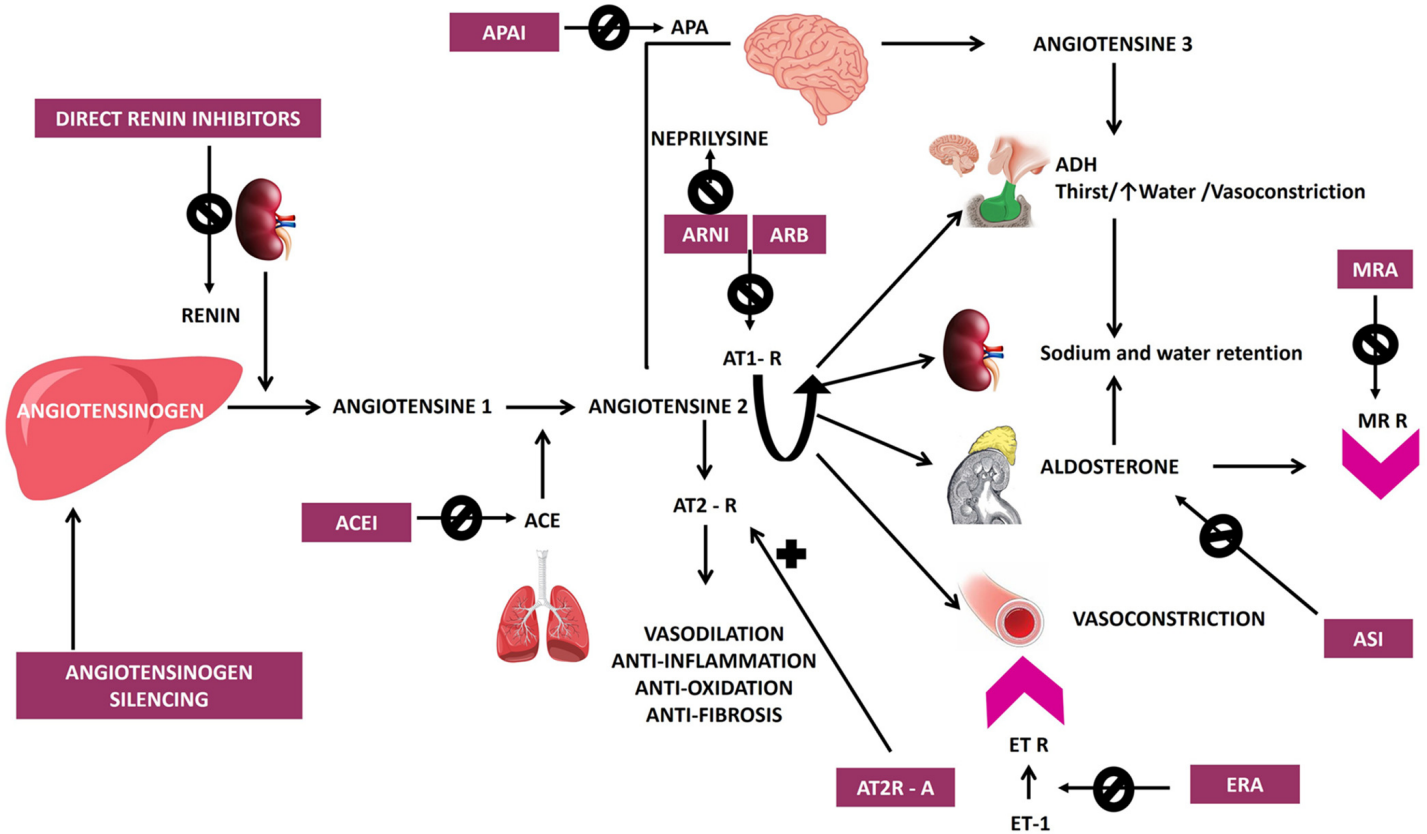
Timeline of antihypertensive drug development from 1950 to 2024, highlighting major therapeutic classes and emerging novel agents, both approved and in phases 1–3 of trials for the treatment of hypertension, including AT_2_R agonists, nsMRA, and various other novel therapeutic approaches. ACEI, angiotensin-converting enzyme inhibitors; APAIs, aminopeptidase A inhibitors; ASI, aldosterone synthase inhibitors; AT_1_R, angiotensin II type 1 receptor blockers; AT_2_R, angiotensin II type 2 receptor agonists; ARNI, angiotensin receptor neprilysin inhibitor; CCB, calcium channel blockers; ERA, endothelin receptor antagonist; GLP1 RAs, glucagon-like peptide 1 receptor agonists; HCZ, hydrochlorothiazide; HTN, hypertension; nsMRA, non-steroidal mineralocorticoid receptor blocker; NPR1 agonists, natriuretic peptide receptor 1 agonists; R, receptor; RAS, renin-angiotensin system; sGC, soluble guanylate cyclase, stimulator; siRNA, small interfering RNA; VAP-1, vascular adhesion protein 1.

Hypertension affects around one billion people worldwide, representing one of the major global health issues (1), adding a striking burden of cardiovascular morbidity and increased mortality. Despite the progress made along the timeline in antihypertensive treatment (Figure 1), a significant number of patients still do not reach the recommended blood pressure (BP) targets (2).

Large-scale epidemiological studies consistently demonstrate that a significant proportion of treated patients fail to reach recommended targets. A serial cross-sectional study of over 18,000 US adults with hypertension found that only 30%–35% of treated individuals achieved blood pressure goals of less than 140/90 mmHg in clinical settings or less than 130/80 mmHg during 24-h ambulatory monitoring (3). Similarly, surveys from Italy involving 52,715 patients showed that, despite improvements in hypertension management, many patients still do not attain adequate control (4). The EURIKA study, conducted across 12 European countries, further underscores this issue, revealing that over half of treated hypertensive patients did not achieve target levels. These findings highlight persistent gaps in current strategies and underscore the need for more effective approaches to primary cardiovascular disease prevention (5).

Moreover, a notable proportion of hypertensive patients experience resistant hypertension, defined as uncontrolled BP, despite being administered at least three different classes of antihypertensive drugs, including a diuretic, all at the maximum tolerated dosages (6, 7).

The development of hypertension is complex and involves multiple pathophysiological factors – excessive activation of the renin-angiotensin-aldosterone system (RAAS), increased activity of the sympathetic nervous system, endothelial dysfunction, and also inflammation (2). Standard antihypertensive drugs often fail to address all these contributing factors effectively, highlighting the necessity to explore new therapeutic targets.

**Figure 2:**
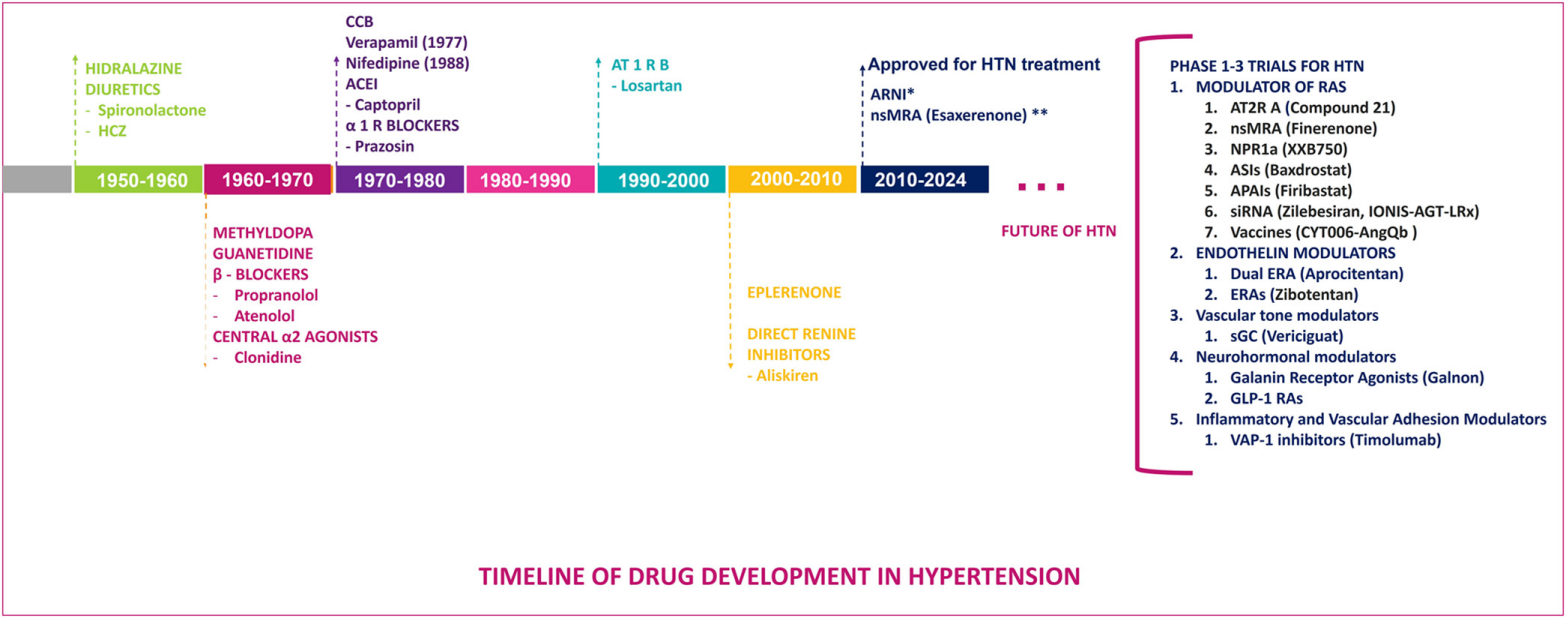
Schematic representation of the renin-angiotensin-aldosterone and endothelin systems along with their pharmacological targets, illustrating classical and novel therapeutic interventions in hypertension including direct renin inhibitors, angiotensin converting enzyme inhibitors (ACEI), angiotensin receptor blockers (ARB), angiotensin receptor neprilysin inhibitors (ARNI) mineralocorticoid receptor antagonists (MRA), and newer agents targeting various pathways, angiotensinogen silencing via siRNA (small interfering RNA), endothelin receptor antagonists (ERA), natriuretic peptide receptor 1 agonists (NPR1 agonists), aminopeptidase A inhibitors (APAIs), aldosterone synthase inhibitors (ASIs). AT_1_R, angiotensin II type 1 receptor; AT_2_R, angiotensin II type 2 receptor; ET R, endothelin receptor; ET, endothelin.

The origins of antihypertensive therapies can be traced back to the 1950s with the introduction of hydralazine and the first thiazide diuretic, chlorothiazide, both of which led the start of pharmacological efforts to manage hypertension, marking the beginning of pharmacological efforts to manage hypertension. Spironolactone, a mineralocorticoid receptor antagonist (MRA), was developed in 1957 and launched in 1959, further expanding treatment options for resistant cases. This was followed in the 1960s by the development of methyldopa and guanethidine, which laid the foundation for subsequent advancements in hypertension treatment. The development of beta-blockers began with propranolol in 1964, while central alpha-2 agonists like clonidine emerged in 1966. The 1970s and 1980s saw significant advancements, including calcium channel blockers (CCBs) such as verapamil in 1977 and nifedipine in 1978, angiotensin-converting enzyme inhibitors (ACEIs) like captopril in 1975 and enalapril in 1981, and alpha-1 receptor blockers like prazosin in 1974. Losartan, the first angiotensin receptor blocker (ARB), was introduced in 1995, marking a pivotal moment in hypertension management due to its selective blockade of angiotensin II type 1 receptors, which effectively reduced BP and cardiovascular risks. The latest innovation came in 2007 with the approval of aliskiren, the first direct renin inhibitor, which targets the RAAS at its rate-limiting step to achieve effective BP control. These milestones reflect continuous progress in developing therapies targeting diverse mechanisms underlying hypertension (Figures 1, 2) (8, 9).

Although pharmaceutical companies have attempted to improve existing drug classes by developing longer-acting agents with fewer side effects and fixed-dose combinations, these innovations have not significantly impacted BP control or prescribing practices on a large scale. Moreover, many individuals with hypertension face challenges related to polypharmacy, which can lead to a range of adverse medication effects, financial strain, and poor adherence to treatment plans (10, 11).

Therefore, there is a critical global need for new approaches for finally overcoming these various barriers, and achieving effective BP management and the good news is there is hope with these new novel therapies that we shall dive deeper into this review.

This review employed a comprehensive search strategy across major databases, including PubMed, Scopus, and Web of Science, from January 2010 to February 2024. The search terms focused on novel antihypertensive therapies, including siRNA-based treatments, CRISPR technology, and other emerging pharmacological agents. Inclusion criteria consisted of studies published in English, addressing hypertension management through these innovative approaches. While the primary focus was on human clinical trials, a few relevant non-human studies were also considered to provide a comprehensive understanding of the mechanisms and potential efficacy of these therapies. Exclusion criteria included abstracts without full-text availability and articles not written in English. The rationale behind these criteria was to ensure that both foundational preclinical evidence and clinical trial data were considered, thereby providing a robust overview of the current state of innovative hypertension treatments. The search results were screened for relevance, and data extraction was performed to summarize key findings related to each therapeutic agent's efficacy, safety profile, and clinical trial progress (also available in Table 1).

**Table 1:** Summary of drugs investigated for hypertension treatment: drug class, mechanisms of action, development/clinical trial status, drug's strengths and weaknesses.

Category	Drug	Main mechanism of action	Clinical trials/status	Strengths	Weaknesses
siRNA Therapies	Zilebesiran	siRNA targeting angiotensinogen to reduce angiotensin II production	Phase 2 (KARDIA-1 trial)	Sustained BP reduction, infrequent dosing, promising safety profile	Limited long-term safety data, high production costs, unclear efficacy vs. combination therapies.
Antisense Oligonucleotides (ASO)	IONIS-AGT-LRx	Antisense oligonucleotide targeting angiotensinogen mRNA	Phase 2	Sustained BP reduction, weekly administration, favorable safety profile	Limited data on long-term efficacy, safety, cost and accessibility, subcutaneous injection administration.
CRISPR-Cas9	No specific CRISPR-Cas9-based drugs have been named yet	Deletion of GPER1 gene, ablation of AGT gene, deletion of AT1R	Preclinical/Early Development	Targeting RAAS components directly at genetic level, bypasses compensatory mechanism. Long-term or lifelong effects.	High costs, ethical considerations, lack of clinical trials. Off-target effects and effective delivery challenges.
Aminopeptidase A Inhibitors (APAIs)	Firibistat	Inhibition of aminopeptidase A to reduce angiotensin III production	Phase 2 NEW-HOPE trial Phase 3 FRESH trial	Potential for reducing BP by targeting RAAS while avoiding common adverse effects of peripheral RAAS inhibitors.	Mixed results from the phase 2 and the phase 3 trials. Cost consideration, long-term safety and efficacy.
	NI956	inhibits brain APA	Preclinical/Early Development	Favorable safety profile	Future clinical trials are needed.
Angiotensin Receptor-Neprilysin Inhibitors (ARNIs)	Sacubitril/Valsartan	Dual inhibition of angiotensin II receptors and neprilysin	Approved in Japan (Phase 3 completed)	Enhanced cardiovascular benefits by combining RAAS and natriuretic peptide pathways	Long-term safety data is needed to address potential risks such as hypotension, hyperkalemia, and renal dysfunction in hypertension
	Sacubitril/allisartan		Phase 3 trial		
Angiotensin II Type 2 Receptor Agonists (AT2R agonists)	Compound 21	Activation of AT2R to counteract AT1R effects	Preclinical/Early Development	Potential for reducing BP, protection against hypertensive end-organ damage.	Need for future clinical trials, also challenges of long-term safety, efficacy, and cost-effectiveness.
Aldosterone Synthase Inhibitors (ASIs)	Baxdrostat	Inhibition of aldosterone synthase (CYP11B2) to reduce aldosterone levels, without affecting cortisol synthesis.	Phase 2 BrigHTN trial	Efficacy in reducing BP, safety profile, not impacting cortisol levels.	Limited clinical data available.
	Lorundrostat		Phase 2 Target-HTN trial Phase 2 Advance-HTN trial Phase 3 Launch-HTN trial	Additional benefits in improving BP control among obese patients, large-scale evidence of efficacy and safety.	Long-term safety and efficacy data needed.
SGLT2 Inhibitors	Empagliflozin, Dapaglifozin, Canaglifozin	Inhibition of glucose reabsorption in the kidney, indirect BP reduction	Not specifically approved for hypertension treatment. Data from clinical trials including patients with diabetes mellitus and heart failure.	Proven efficacy in reducing BP and cardiovascular risk	Potential for increased risk of genital infections and diabetic ketoacidosis. Need for focus on antihypertensive effects in clinical trials.
Non-Steroidal Mineralocorticoid Receptor Antagonists (nsMRAs)	Esaxerenone	Blockade of mineralocorticoid receptors to reduce BP	Phase 3 trial	Potential for reducing BP without steroid-related side effects, while offering additional benefits such as renoprotection and cardiovascular risk reduction.	Side effects as hyperkalemia, but manageable.
	Finerenone		Data from clinical trials including patients with diabetes mellitus and chronic kidney disease or with heart failure.		Need for focus on antihypertensive effects in clinical trials.
	Aparenone		Phase 2 trial		Long-term efficacy to be evaluated further.
Natriuretic peptide receptor 1 agonists (NPR1 agonists)	XXB750	Degradation of mineralocorticoid receptors by targeting NPR1	Phase 2 trial	Once-monthly administration. Avoids hormonal side effects.	Subcutaneous administration issues. Cost potential issue. Efficacy may be limited for targeted population.
Endothelin Antagonists	Aprocitentan	Dual endothelin receptor antagonist	Approved (Phase 3 PRECISION trial completed)	Efficacy in reducing blood pressure in patients with resistant hypertension. Sustained effect.	Limited Dose-Response. Potential for fluid retention and other side effects. Contraindicated in pregnancy.
	Zibotentan	Endothelin A receptor selective antagonist	Data from clinical trials including patients with chronic kidney disease (ZENITH-CKD trial) and microvascular angina (PRIZE trial).	Proven efficacy in reducing BP in certain conditions.	Need for focus on antihypertensive effects in clinical trials.
Soluble Guanylate Cyclase Stimulators (sGCs)	Vericiguat	Enhances the cGMP pathway by directly stimulating sGC	Data from clinical trials including patients with heart failure.	Proven efficacy in reducing BP in certain conditions	Need for focus on antihypertensive effects in clinical trials.
Galanin Receptor Agonists	Galnon (synthetic non-peptide)	Activation of galanin receptors to reduce sympathetic activity	No specific information available indicating development for HTN.	Potential for reducing BP by targeting sympathetic nervous system	Its primary applications are not focused on hypertension treatment.
GLP-1 Receptor Agonists (GLP-1 RAs)	Liraglutide, semaglutide, dulaglutide, and tirzepatide	Activation of GLP-1 receptors, inhibition of RAAS.	Data from clinical trials including patients with diabetes or obesity.	Modest reduction of BP, reduces metabolic and cardiovascular outcomes	Potential for gastrointestinal side effects. Limited clinical data available.
Vascular Adhesion Protein-1 (VAP-1) Inhibitors	Timolumab	Inhibition of VAP-1 and enhancing endothelial function	Data from clinical trials including patients with inflammatory conditions.	Potential for reducing BP. Good safety profile.	Limited clinical data available.
Hypertension vaccines	AngQb-Cyt006	Stimulates the production of anti-angiotensin II antibodies using virus-like particles conjugated with angiotensin II peptides.	Phase IIa trials completed	Demonstrated significant reductions in blood pressure in Phase IIa trials. Well-tolerated with no serious adverse events.	Lower efficacy compared to existing RAS inhibitors. Development was discontinued due to this lower efficacy.
	AGMG0201	Uses a DNA platform to induce long-lasting antibody responses against angiotensin II.	Phase I/IIa	Induces measurable anti-Ang II antibody titers. Confirmed safety and tolerability.	Requires further clinical studies to assess antihypertensive effects.
	ATRQβ-001	Utilizes peptide fragments derived from the angiotensin II type 1 receptor conjugated with Qβ bacteriophage virus-like particles.	Preclinical/Early Development	Demonstrated significant blood pressure reductions in preclinical studies. Prevented target organ damage in animal models.	Requires further development to assess its potential in humans.

AT1R, angiotensin type 1 receptor; BP, blood pressure; HTN, hypertension; RAAS, renine angiotensine aldosterone system; SGLT2i, sodium glucose co-transporter 2 inhibitors; siRN, small interfering ribonucleic acid.

## 2 Emerging therapeutic frontiers in hypertension management

### 2.1 Modulators of the renin-angiotensin-aldosterone system (RAAS)

#### 2.1.1 Angiotensinogen silencing via siRNA

Gene-based therapies, particularly those utilizing RNA-based approaches and CRISPR-Cas9, represent a transformative frontier in hypertension management. These technologies aim to modify the underlying genetic contributors to hypertension, offering the potential for long-term or even curative effects.

Zilebesiran and IONIS-AGT-LRx represent groundbreaking advancements in the field of small interfering RNA (siRNA) therapeutics, specifically designed to target and silence angiotensinogen expression. By effectively reducing the production of angiotensin II, these agents proved their associated hypertensive effects, offering a novel mechanism of action in the management of hypertension (10, 12).

Early clinical trials have shown promising results, demonstrating significant BP reductions, thereby underscoring their potential as future antihypertensive agents.

A first-in-class siRNA therapeutic, **zilebesiran** operates by binding to the hepatic asialoglycoprotein receptor, resulting in a significant reduction in angiotensinogen messenger RNA (mRNA), subsequently decreasing hepatic production of angiotensinogen. The suppression of angiotensinogen synthesis leads to a downstream reduction in both angiotensin I and angiotensin II levels, ultimately leading to a lowered BP (12, 13).

Zilebesiran has demonstrated significant and sustained reductions in BP across multiple clinical trials, positioning it as a promising therapeutic option for hypertension management. In the Phase 1 trial conducted by Desai et al., zilebesiran showed dose-dependent reductions in serum angiotensinogen levels and 24-h ambulatory systolic BP (SBP). At doses of 200 mg or higher, patients experienced a mean reduction of 10 mmHg in SBP and 5 mmHg in DBP at 8 weeks, with effects sustained for up to 24 weeks. Notably, the highest dose of 800 mg achieved reductions in SBP of up to 20 mmHg at six months, with no serious adverse events reported. Mild injection-site reactions were observed in five patients, but there were no cases of hypotension, hyperkalemia, or renal dysfunction requiring intervention (13).

However, prolonged RAAS suppression raises theoretical concerns in acute conditions where RAAS activation is critical for hemodynamic stability, such as severe hypotension, hemorrhage, or septic shock. While clinical trials to date have not reported such complications, long-term safety monitoring in diverse populations is warranted to assess these risks.

Further evidence from the KARDIA-1 Phase 2 trial reinforced these findings. The study enrolled 394 adults with mild-to-moderate hypertension and randomized them to receive varying doses of zilebesiran (150 mg, 300 mg, or 600 mg) or placebo. At three months, the trial met its primary endpoint, showing a placebo-adjusted reduction in SBP of over 15 mmHg with both the 300 mg and 600 mg doses (*p* < 0.0001). These reductions persisted through six months, supporting quarterly or biannual dosing regimens. Secondary endpoints also demonstrated consistent reductions in office SBP and DBP at three and six months across all zilebesiran arms (14–16).

When compared to standard antihypertensive therapies such as ACE inhibitors or ARBs, zilebesiran offers the advantage of infrequent dosing while maintaining sustained BP control. This could address adherence challenges commonly associated with daily oral medications. However, long-term safety data are still limited, particularly regarding potential off-target effects or adverse events over extended periods (17).

Barriers to clinical adoption include high production costs associated with RNA interference (RNAi) therapeutics and accessibility challenges, particularly in low-resource settings. Furthermore, while zilebesiran demonstrates superior BP reductions compared to placebo, its efficacy relative to combination therapies involving multiple antihypertensive agents remains unclear.

**IONIS-AGT-LRx**, an antisense oligonucleotide (ASO) targeting angiotensinogen (AGT) mRNA, represents a novel therapeutic strategy for hypertension management by inhibiting the RAAS upstream at the hepatic level. In Phase 1 trials, IONIS-AGT-LRx demonstrated significant reductions in plasma AGT levels, with a decrease of up to 60% in the highest single-dose cohort (80 mg) and 54% in multiple-dose cohorts compared to placebo. These reductions persisted for several weeks post-treatment, highlighting the durability of its effect. Importantly, no significant adverse events were observed, including no cases of acute kidney injury (AKI), hyperkalemia, or liver dysfunction, underscoring its favorable safety profile (17, 18).

In Phase 2 trials, IONIS-AGT-LRx was evaluated both as monotherapy and as an add-on therapy in patients with hypertension. In one trial involving patients with uncontrolled hypertension already on 2–3 antihypertensive medications, weekly subcutaneous administration of IONIS-AGT-LRx for six weeks resulted in a mean reduction of plasma AGT levels by 69%, coupled with a significant reduction in SBP by 10.6 mmHg and DBP by 7.3 mmHg compared to placebo (18). Another Phase 2 study in patients with mild hypertension reported a mean SBP reduction of approximately 8 mmHg, although these trials were not powered to detect BP changes as primary endpoints (19).

Compared to conventional RAAS inhibitors like ACE inhibitors or ARBs, IONIS-AGT-LRx offers a more upstream mechanism of action by directly reducing AGT production. This approach may provide more comprehensive RAAS inhibition while potentially avoiding compensatory increases in renin or angiotensin II levels often seen with ACE inhibitors and ARBs. However, unlike zilebesiran, which has demonstrated robust BP reductions as a primary endpoint in clinical trials, IONIS-AGT-LRx has shown only trends toward BP reduction thus far, suggesting partial RAAS blockade (17, 18).

Despite its promise, several limitations and barriers to clinical adoption remain. The need for weekly subcutaneous injections may pose challenges for patient adherence compared to oral antihypertensive therapies. Additionally, long-term safety data are limited, particularly regarding potential off-target effects or adverse events over extended treatment periods. Cost and accessibility are also concerns, as ASO therapies are typically expensive to produce and may not be readily available in resource-limited settings. Furthermore, adherence to infrequent dosing schedules (e.g., quarterly or biannual injections) requires robust patient education and follow-up systems (17, 18).

#### 2.1.2 CRISPR-Cas9

CRISPR-Cas9 technology has emerged as a promising therapeutic tool for hypertension by targeting genetic contributors to BP regulation. While still in the experimental phase, preclinical studies have demonstrated significant potential for this approach to address the root causes of hypertension (19).

The AGT gene, a key component of the RAAS, has been successfully targeted using CRISPR-Cas9 in animal models. In one study, partial ablation (∼40%) of AGT expression in spontaneously hypertensive rats (SHRs) resulted in sustained reductions in SBP by approximately 30 mmHg over three months. Importantly, this intervention did not impair cardiovascular stress responses, highlighting its safety profile (20).

CRISPR-Cas9-mediated deletion of the GPER1 gene in salt-sensitive hypertensive rats led to statistically significant reductions in SBP (by 17 mmHg) and DBP (by 25 mmHg). This approach modulated vascular tone through microbiota-dependent mechanisms, demonstrating its potential to address vascular dysfunction (21).

Deletion of the AT1R gene using CRISPR-Cas9 in hypertensive mice resulted in marked reductions in BP and improved cardiovascular function. This study provided proof-of-concept for targeting RAAS components directly at the genetic level (22).

Gene editing offers long-lasting effects, potentially eliminating the need for daily medication adherence, and by addressing genetic contributors directly, CRISPR-Cas9 bypasses compensatory mechanisms often seen with pharmacological RAAS inhibitors, providing long-term of lifelong effects (23).

Currently, CRISPR-Cas9 therapies for hypertension remain in preclinical stages, with no therapeutic agents approved for clinical use. Early-phase clinical trials are anticipated to evaluate safety and efficacy, focusing on minimizing off-target effects and optimizing delivery systems, as off-target effects could lead to unintended genetic modifications, posing risks such as tumorigenesis or organ dysfunction. Moreover, effective delivery of CRISPR-Cas9 components to the target tissues remains a technical hurdle (24).

Furthermore, high costs associated with CRISPR technology may limit its availability, particularly in low-resource settings, and ethical considerations need to be addressed as well, as germline editing raises serious ethical concerns.

#### 2.1.3 Aminopeptidase A inhibitors (APAIs)

Angiotensin III contributes to elevated BP by stimulating vasopressin release, increasing sympathetic nervous system activity, enhancing vascular resistance, and activating baroreflex mechanisms. Aminopeptidase A (APA), the enzyme responsible for converting angiotensin II to angiotensin III, represents a promising therapeutic target for central RAAS inhibition (25).

**Firibastat**, a first-in-class aminopeptidase A inhibitor (APAI), reduces angiotensin III production, effectively lowering BP while avoiding common adverse effects of peripheral RAAS inhibitors, such as hyperkalemia and renal impairment (26).

The efficacy of firibastat was demonstrated in the NEW-HOPE trial, an open-label Phase 2 study involving 256 hypertensive patients from diverse ethnic backgrounds, including 54% Black and Hispanic individuals. After eight weeks of treatment with firibastat (250 mg twice daily for two weeks, followed by 500 mg twice daily for six weeks), systolic automated office BP (AOBP) decreased by 9.5 mmHg (*p* < 0.0001), and diastolic AOBP decreased by 4.2 mmHg (*p* < 0.0001). Subgroup analyses revealed greater reductions in SBP among Black patients (−10.5 mmHg) and obese patients (−10.2 mmHg). Importantly, no significant changes in potassium, sodium, or creatinine levels were observed, and adverse events were limited to mild headaches (4%) and skin reactions (3%) (27).

However, the Phase 3 Firibastat in Treatment-resistant Hypertension (FRESH) trial yielded disappointing results. This double-blind, placebo-controlled study enrolled 515 participants across 75 sites in 11 countries, including individuals with difficult-to-treat hypertension (SBP 140–179 mmHg despite treatment with at least two antihypertensive classes) and resistant hypertension (SBP ≥140 mmHg despite treatment with at least three classes, including a diuretic). Participants received firibastat (500 mg twice daily) or placebo for three months. The primary endpoint—change in unattended clinic SBP—showed no significant difference between the firibastat group and placebo (−7.8 mmHg vs. −7.9 mmHg; *p* = 0.98). Similarly, secondary endpoints such as DBP and 24-h ambulatory BP did not differ significantly between groups, raising questions about firibastat's efficacy in resistant hypertension (28).

Despite these mixed results, firibastat's novel mechanism of action highlights its potential in addressing hypertension in populations less responsive to conventional therapies, such as those with low renin activity or salt sensitivity. Limitations of current studies include small sample sizes in early trials and the lack of head-to-head comparisons with standard antihypertensive agents like ACE inhibitors or ARBs. Additionally, barriers to clinical adoption include cost considerations associated with novel therapeutics and uncertainties regarding long-term safety and efficacy (29).

While firibastat represents an innovative approach to targeting central RAAS mechanisms through APA inhibition, further research is needed to identify patient subgroups most likely to benefit from this therapy and to address its limitations in resistant hypertension management (30).

In addition to firibastat, other APAIs have been developed and investigated for their potential in hypertension management. **NI956** (also known as QGC006) is another orally active, brain-penetrating APAI prodrug that has shown promising results in preclinical studies. NI956 inhibits brain APA activity, reduces angiotensin III formation, and lowers BP by decreasing plasma arginine-vasopressin (AVP) levels and increasing diuresis and natriuresis. In DOCA-salt hypertensive rats, NI956 demonstrated a significant reduction in mean arterial BP without affecting plasma potassium levels, further supporting its safety profile (31).

#### 2.1.4 Angiotensin receptor-neprilysin inhibitors (ARNIs)

**Sacubitril/valsartan**, the first angiotensin receptor-neprilysin inhibitor (ARNI), has demonstrated significant antihypertensive effects and cardiovascular benefits in clinical trials, particularly in patients with heart failure (HF) and hypertension.

In the PARADIGM-HF trial, sacubitril/valsartan reduced cardiovascular mortality and hospitalization in HF with reduced ejection fraction (HFrEF) by 20% compared to enalapril, establishing its role in HFrEF management (32, 33).

While the PARAGON-HF trial in HF with preserved ejection fraction (HFpEF) did not meet its primary endpoint, subgroup analyses suggested benefits in patients with left ventricular ejection fraction (LVEF) <57% and in women (34–36). Pooled data from both trials further indicated reduced hospitalizations in patients with LVEF <60% (37). These findings highlight ARNIs’ potential in hypertensive patients with overlapping HF, particularly given their dual mechanism of RAAS inhibition and natriuretic peptide enhancement.

A *post hoc* analysis from the PARAGON-HF trial demonstrated that sacubitril/valsartan achieved BP control in 47.9% of patients with apparent resistant hypertension compared to 34.3% treated with valsartan alone [adjusted odds ratio (OR) 1.78; CI: 1.30–2.43]. In patients with mineralocorticoid receptor antagonist-resistant hypertension, BP control was achieved in 43.6% vs. 28.4% of patients treated with valsartan alone (adjusted OR 2.63; CI: 1.18–5.89) (38).

In a meta-analysis including seven studies and over 3,300 patients, sacubitril/valsartan significantly reduced sitting SBP by −4.70 mmHg (CI: −5.79 to −3.61; *p* < 0.001) and DBP by −2.29 mmHg (CI: −2.53 to −2.04; *p* < 0.001) compared to ARBs alone (39).

Sacubitril/valsartan has been shown to effectively control resistant hypertension in hemodialysis patients. A study by Wang et al. demonstrated that sacubitril/valsartan significantly reduced mean sitting systolic and diastolic blood pressure by −20.7 mmHg and −8.3 mmHg, respectively, in patients with resistant hypertension. Additionally, it improved myocardial work and life quality without serious adverse events (40).

Furthermore, a small trial in Japanese patients with mild-to-moderate hypertension demonstrated that sacubitril/valsartan reduced SBP by approximately −5 mmHg more than olmesartan (41), leading to its approval for hypertension treatment in Japan in 2021 (42).

Despite its promising results, barriers to clinical adoption include cost considerations and accessibility challenges, particularly in low-resource settings. Long-term safety data is needed to address potential risks such as hypotension, hyperkalemia, and renal dysfunction observed in some trials. Additionally, while sacubitril/valsartan offers superior BP control compared to RAAS inhibitors, its role in reducing cardiovascular outcomes beyond HF populations requires further validation.

Besides sacubitril/valsartan, other ARNIs are being investigated for the treatment of hypertension. Sacubitril/allisartan, a novel angiotensin receptor-neprilysin inhibitor (ARNI), has shown significant potential in the treatment of hypertension based on findings from Phase 2 and Phase 3 clinical trials. In a Phase 3 randomized, double-blind, multicenter trial involving 1,197 Chinese patients with mild-to-moderate hypertension, sacubitril/allisartan demonstrated dose-dependent reductions in clinic mean sitting systolic blood pressure (msSBP) over 12 weeks. At the highest dose of 480 mg/day, sacubitril/allisartan reduced msSBP by −28.2 mmHg compared to −23.2 mmHg with olmesartan 20 mg/day (between-group difference: −5.0 mmHg; 95% CI: −7.3 to −2.8; *p* < 0.001). Similarly, the 240 mg/day dose achieved a reduction of −25.1 mmHg, which was non-inferior to olmesartan (difference: −1.9 mmHg; 95% CI: −4.2 to −0.4; *p* = 0.0007). Significant reductions in DBP and 24-h ambulatory BP were also observed, with greater effects on nighttime BP compared to olmesartan (*p* ≤ 0.001) (43). The magnitude of BP reduction in this trial is remarkable and makes this ARNI a promising tool in achieving hypertension control. However, sacubitril/allisartan faces several challenges: as a novel ARNI, it may be more expensive than traditional antihypertensive therapies like ACE inhibitors or ARBs; although short-term efficacy is well established, long-term cardiovascular outcomes remain unknown; and its availability may as well be limited in low-resource settings.

#### 2.1.5 Angiotensin II type 2 receptor agonists (AT2R)

Angiotensin II type 2 receptor (AT2R) agonists represent a therapeutic class with potential applications in resistant hypertension and other cardiovascular conditions. Unlike angiotensin II type 1 receptor (AT1R) blockers, which primarily inhibit vasoconstriction and sodium retention, AT2R agonists selectively stimulate the AT2 receptor to counteract AT1R-mediated effects. These include promoting vasodilation, anti-inflammatory responses, and tissue protection (44).

Studies in spontaneously hypertensive rats demonstrated that the selective AT2R agonist **CGP42112** only reduced BP when co-administered with a low dose of the AT1R antagonist candesartan, suggesting that AT2R-mediated vasodilation becomes apparent only when background AT1R stimulation is attenuated. This synergistic effect highlights the potential therapeutic value of combining AT2R agonists with low-dose AT1R blockers in specific cardiovascular conditions (45).

The leading AT2R agonist, **Compound 21 (C21)**, has demonstrated significant potential in preclinical and early clinical studies. Preclinical data in stroke-prone spontaneously hypertensive rats (SHRs) and Dahl salt-sensitive rats have shown that C21 reduces vascular injury, myocardial fibrosis, renal inflammation, and end-organ damage. In SHRs, chronic administration of C21 reduced SBP by approximately 15 mmHg compared to controls (*p* < 0.05). Additionally, C21 promoted natriuresis and diuresis while exerting anti-inflammatory effects through the suppression of pro-inflammatory cytokines such as interleukin-6 and tumor necrosis factor-alpha (*p* < 0.01) (46).

However, experimental evidence indicates that systemic administration of AT2R agonists is largely BP-neutral, but targeted stimulation of AT2R within specific organs such as the brain or kidneys can elicit systemic BP-lowering effects. Additionally, AT2R stimulation can achieve antihypertensive effects when combined with low-dose AT1R blockade, which attenuates the background AT1R stimulation. Despite the minimal direct impact on BP when used alone, AT2R agonists demonstrate significant protection against hypertensive end-organ damage (47).

While representing a promising advancement in hypertension management by selectively targeting AT2Rs to provide vasodilatory, anti-inflammatory, and tissue-protective effects, further research with clinical trials is required to establish its role in human hypertension control, alongside long-term safety, efficacy, cost-effectiveness, and role in combination therapies.

#### 2.1.6 Aldosterone synthase inhibitors (ASIs)

Aldosterone synthase inhibitors (ASIs) represent a novel drug class that directly reduces circulating aldosterone levels by inhibiting CYP11B2, the enzyme responsible for aldosterone synthesis while preserving cortisol production (48).

Compared to MRAs, ASIs offer several advantages. While MRAs block aldosterone receptors and can lead to compensatory increases in aldosterone, potentially activating harmful non-genomic pathways, ASIs suppress aldosterone production at its source, thus avoiding these effects. ASIs are also less likely to cause side effects such as hyperkalemia, gynecomastia, or sexual dysfunction, since they selectively inhibit CYP11B2 without interfering with other steroid receptors (48).

Baxdrostat (CIN-107), a highly selective ASI, has demonstrated remarkable efficacy in the Phase 2 BrigHTN trial. This multicenter, randomized, double-blind, placebo-controlled study enrolled 275 patients with treatment-resistant hypertension who were already receiving at least three antihypertensive medications. Participants were randomized to receive once-daily baxdrostat (0.5 mg, 1 mg, or 2 mg) or a placebo for 12 weeks. The trial revealed dose-dependent reductions in SBP: −20.3 mmHg, −17.5 mmHg, −12.1 mmHg, and −9.4 mmHg in the 2-mg, 1-mg, 0.5-mg, and placebo groups, respectively. The placebo-adjusted reduction in the 2-mg group was −11.0 mmHg (*p* < 0.0001) and −8.1 mmHg (*p* = 0.0030) in the 1-mg group. Notably, approximately 46% of patients in the 2-mg dose arm achieved BP control (SBP less than 130 mmHg). Baxdrostat also significantly lowered DBP by 5.2 mmHg in the 2-mg dose group compared to placebo (49).

Regarding safety, baxdrostat demonstrated a favorable profile with no serious adverse events attributed to the medication (49, 50). In early Phase 1 studies, baxdrostat showed a dose-dependent reduction of plasma aldosterone by approximately 51%–73% after 10 days of treatment, with no meaningful impact on plasma cortisol levels. The half-life of 26–31 h supports once-daily dosing. In the BrigHTN trial, all treatment-emergent adverse events in subjects receiving baxdrostat were mild in severity (49, 50).

Despite these promising results, several limitations exist. The trial excluded patients with severe hypertension (SBP ≥180 mmHg or DBP ≥110 mmHg), limiting generalizability to this high-risk population. Additionally, baxdrostat's effectiveness was only evaluated against placebo rather than active comparators like mineralocorticoid receptor antagonists (MRAs).

Baxdrostat is currently advancing in clinical development, with results expected to further establish its role in the hypertension treatment paradigm (51).

**Lorundrostat**, another selective ASI, has demonstrated significant efficacy and safety in clinical trials for the treatment of uncontrolled and resistant hypertension. By targeting CYP11B2, the enzyme responsible for aldosterone synthesis, lorundrostat reduces circulating aldosterone levels while preserving cortisol production, offering a targeted mechanism of action for hypertension management (52).

The Phase 2 Target-HTN trial was a randomized, double-blind, placebo-controlled, dose-ranging study conducted in 200 patients with uncontrolled or resistant hypertension on at least two background antihypertensive medications (53). Participants were randomized to receive lorundrostat at doses of 12.5 mg once daily (QD), 50 mg QD, 100 mg QD, or placebo for 12 weeks. The trial met its primary endpoint, demonstrating statistically significant reductions in SBP. Lorundrostat 50 mg QD achieved a placebo-adjusted reduction in SBP of −9.6 mmHg (*p* = 0.01), while the 100 mg QD dose achieved a reduction of −7.8 mmHg (*p* = 0.04). The BP-lowering effects were particularly pronounced in patients with obesity, likely due to visceral fat driving abnormal aldosterone levels (54).

The Phase 3 Launch-HTN trial further confirmed these findings. This trial enrolled 1,083 patients with uncontrolled or resistant hypertension despite being on two to five antihypertensive medications. At week 6, lorundrostat 50 mg QD resulted in a placebo-adjusted SBP reduction of −9.1 mmHg (*p* < 0.0001). By week 12, the placebo-adjusted reductions from baseline were −11.7 mmHg (*p* < 0.0001) for the 50 mg group and −8.4 mmHg (*p* = 0.0016) for the group that escalated from 50 mg to 100 mg after week 6 (3). These reductions were consistent across both uncontrolled and resistant hypertension subgroups (55).

The Phase 2 Advance-HTN trial also demonstrated significant BP reductions using ambulatory BP monitoring (ABPM). At week 12, lorundrostat achieved a placebo-adjusted reduction in SBP of −7.9 mmHg (*p* < 0.0001), further supporting its efficacy as an add-on therapy for hypertension management (56).

Both lorundrostat and baxdrostat target CYP11B2 to reduce aldosterone levels and exhibit similar BP-lowering efficacy. However, lorundrostat has shown additional benefits in improving BP control rates among obese patients, likely due to its impact on visceral fat-driven aldosterone dysregulation.

Despite promising results, several challenges remain. Lorundrostat is not yet FDA-approved for hypertension treatment but is advancing through Phase 3 trials. The discussion of cost needs to be addressed as well. ASIs are likely to be more expensive than conventional therapies like ACE inhibitors or ARBs. Moreover, long-term cardiovascular outcomes are still under investigation, and potassium levels require careful monitoring during treatment (57).

With positive results from pivotal trials like Target-HTN, Launch-HTN, and Advance-HTN, lorundrostat is advancing toward potential regulatory approval and may offer a transformative option for millions of patients inadequately controlled by existing therapies (53).

#### 2.1.7 Sodium-glucose cotransporter 2 inhibitors (SGLT2i)

Sodium-glucose cotransporter 2 inhibitors (SGLT2i), including **empagliflozin, dapagliflozin, and canagliflozin**, have emerged as a promising class of medications with some antihypertensive effects, in addition to their established benefits in type 2 diabetes mellitus (T2DM) and HF (58–60). While not specifically approved for hypertension treatment, SGLT2i demonstrate consistent BP-lowering effects that warrant consideration in patients with comorbid conditions.

By inhibiting glucose reabsorption in the proximal tubule, SGLT2i induce glucosuria, leading to osmotic diuresis and sodium excretion. This reduces plasma volume and cardiac preload, contributing to acute BP lowering (58). SGLT2i downregulate sympathetic nervous system (SNS) hyperactivity and attenuate RAAS activation. Dapagliflozin reduces norepinephrine levels and angiotensin II receptor expression, while canagliflozin decreases aldosterone synthesis (59). Chronic use improves arterial stiffness and endothelial function via reduced oxidative stress and inflammation. Empagliflozin, for example, enhances nitric oxide bioavailability and suppresses endothelin-1, improving vascular compliance (60).

A comprehensive meta-analysis of 10 randomized controlled trials involving 9,913 participants revealed that SGLT2i significantly reduced 24-h systolic blood pressure by 5.06 mmHg [95% CI (−7.10, −3.01), *p* < 0.05] and 24-h diastolic blood pressure by 2.39 mmHg [95% CI (−4.11, −0.67), *p* = 0.004] compared to placebo. Office systolic and diastolic BP measurements also showed significant reductions of 4.53 mmHg [95% CI (−5.66, −3.40), *p* < 0.05] and 2.12 mmHg [95% CI (−3.42, −0.82), *p* = 0.001], respectively (61).

Empagliflozin reduced SBP by −5.49 mmHg and DBP by −6.19 mmHg after 12 weeks in hypertensive patients with T2DM and showed sustained effects in ambulatory BP monitoring (ABPM) (62).

Similarly, dapagliflozin reduced SBP by −3.6 mmHg and DBP by −1.2 mmHg over 24 weeks (63), while canagliflozin achieved a placebo-adjusted reduction of −3.50 mmHg in SBP within three weeks, maintained throughout the CREDENCE trial (63, 64).

The antihypertensive effects of SGLT2i appear to be independent of their glycemic control benefits. SGLT2i promote weight loss, particularly visceral adiposity, which is linked to improved insulin sensitivity and reduced SNS-driven hypertension. A recent study suggests that the BP-lowering effect of SGLT2i may be more pronounced in non-obese patients, highlighting the potential for targeted therapy (65).

Furthermore, in a meta-analysis of 10 randomized controlled trials, SGLT2i have demonstrated superiority over dipeptidyl peptidase-4 (DPP-4) inhibitors, one of the most commonly used antidiabetic agents, in reducing the risk of incident hypertension in patients with diabetes without prior known diagnostic of hypertension, suggesting a better potential preventive role than DPP-4 inhibitors use (66).

Despite these promising findings, several limitations and barriers to the adoption of SGLT2i for primary hypertension management exist, including cost considerations, potential side effects such as genital infections, and the need for long-term safety data in non-diabetic hypertensive populations. Additionally, without specific FDA approval for hypertension, insurance coverage may be limited for patients without comorbid conditions like diabetes or HF.

SGLT2 inhibitors show significant promise as adjunctive agents for hypertension management, particularly in patients with comorbid conditions such as diabetes, HF, or CKD. While their antihypertensive effects are well-documented in clinical trials the magnitude of BP reduction is still modest. Therefore, their implementation as primary antihypertensive agents is doubtable (67).

#### 2.1.8 Non-steroidal mineralocorticoid receptor antagonists (nsMRAs)

Traditional steroid-based mineralocorticoid receptor antagonists (sMRAs), such as spironolactone and eplerenone, are effective in managing hypertension but are limited by side effects, including hyperkalemia, gynecomastia, and renal dysfunction, which often affect patient compliance and limit their long-term use. Non-steroidal mineralocorticoid receptor antagonists (nsMRAs), such as finerenone and esaxerenone, have emerged as promising alternatives with enhanced safety profiles and efficacy (68).

Esaxerenone has made significant advancements in hypertension management and is approved in Japan for the treatment of essential hypertension (69). The Phase 2 trial (NCT02890173) demonstrated dose-dependent reductions in BP. At doses of 1.25 mg, 2.5 mg, and 5 mg daily, esaxerenone reduced sitting SBP by −10.7 mmHg, −14.3 mmHg, and −20.6 mmHg, respectively, compared to −7.0 mmHg with placebo (*p* < 0.001 for all doses). The reductions in DBP ranged from −5.0 mmHg to −10.4 mmHg (*p* < 0.0001) compared to placebo. Esaxerenone also showed efficacy comparable to eplerenone but with a longer half-life, providing sustained BP control over a 24-h dosing window (70, 71).

A long-term Phase 3 study further confirmed the efficacy of esaxerenone as monotherapy or combined with RAAS inhibitors or calcium channel blockers. Over 52 weeks, esaxerenone reduced sitting SBP by −23.1 mmHg and DBP by −12.5 mmHg (*p* < 0.0001), with consistent reductions across patient subgroups stratified by age and baseline BP levels (72). Hyperkalemia was reported in 5.4% of patients but was manageable with dietary modifications.

**Finerenone** is another nsMRA that has demonstrated significant promise in clinical trials due to its high selectivity for the mineralocorticoid receptor (MR) and reduced risk of hyperkalemia compared to sMRAs. The FIDELIO-DKD trial, a Phase 3 study involving patients with chronic kidney disease (CKD) and type 2 diabetes mellitus (T2DM), confirmed finerenone's ability to modestly lower BP while providing renoprotective benefits (73, 74) and lowering albuminuria (75).

In the FINEARTS-HF trial, finerenone was evaluated in patients with heart failure with mildly reduced or preserved ejection fraction (HFmrEF/HFpEF). Finerenone significantly reduced the composite risk of cardiovascular death or total HF events compared to placebo while demonstrating modest BP-lowering effects in some patients (*p* < 0.05). Safety outcomes showed a slightly higher incidence of SBP <100 mmHg in the finerenone group compared to placebo (18.5% vs. 12.4%), but overall tolerability was favorable (76).

Compared to sMRAs like spironolactone or eplerenone, nsMRAs such as esaxerenone and finerenone offer several advantages. nsMRAs exhibit lower risks of hyperkalemia and sex hormone-related adverse effects due to their higher selectivity for MR. Both agents provide sustained BP control while offering additional benefits such as renoprotection and cardiovascular risk reduction. Esaxerenone has demonstrated stable antihypertensive effects over a year-long treatment period.

However, barriers to clinical adoption include cost considerations, particularly for finerenone, which is FDA-approved for cardiorenal protection but not specifically for hypertension treatment outside CKD/T2DM populations. Hyperkalemia remains a concern for both agents, though it is less frequent than with sMRAs.

The development and approval of finerenone and esaxerenone represent a pivotal shift in hypertension management, providing enhanced efficacy and safety profiles that address the limitations of older MRAs. Ongoing research and future clinical trials are expected to further clarify their roles in treating resistant hypertension and their potential benefits across broader patient populations.

Besides finerenone and esaxerenone, other non-steroidal mineralocorticoid receptor antagonists (nsMRAs) are currently under investigation for their potential in hypertension and related cardiovascular conditions.

Apararenone is a selective nsMRA that has demonstrated antihypertensive effects and renal protection in Phase 2 clinical trials (77). In a study involving patients with CKD and hypertension, apararenone significantly reduced SBP by −10.2 mmHg (*p* < 0.001) compared to placebo over 12 weeks. It also lowered albuminuria by 30% (*p* < 0.01), highlighting its renoprotective benefits. Apararenone exhibited a favorable safety profile, with fewer incidences of hyperkalemia compared to steroidal MRAs like spironolactone (78). However, long-term efficacy and cardiovascular outcomes remain to be evaluated in Phase 3 trials.

#### 2.1.9 Natriuretic peptide receptor 1 agonists (NPR1 agonists)

**XXB750**, a first-in-class natriuretic peptide receptor 1 (NPR1) agonist developed by Novartis, is an investigational monoclonal antibody designed to activate NPR1. NPR1 activation plays an important role in BP regulation by mediating the vasodilatory, natriuretic, and diuretic effects of atrial natriuretic peptide (ANP) and brain natriuretic peptide (BNP) (79). By directly binding to and activating NPR1, XXB750 promotes cyclic guanosine monophosphate (cGMP) synthesis, bypassing the RAAS and offering a novel therapeutic approach for resistant hypertension and HFrEF.

XXB750 has been evaluated in Phase 1 and Phase 2 clinical trials. The Phase 1 trial (NCT05328752), a randomized, double-blind, placebo-controlled study, investigated its safety and tolerability in patients with HFrEF and heart failure with HFmrEF. The trial included two cohorts: Cohort 1 received a single dose of XXB750 or placebo, while Cohort 2 received multiple doses over 56 days with a follow-up period of 13 weeks. The study demonstrated that XXB750 was generally well-tolerated, with no major safety concerns reported (80).

The ongoing Phase 2 trial (81) is evaluating XXB750's efficacy in resistant hypertension. This multi-center, randomized, double-blind, dose-finding study involves patients with resistant hypertension who are already on at least three antihypertensive medications. Preliminary results indicate that XXB750 reduces mean 24-h ambulatory SBP by approximately −15 mmHg at the highest dose of 240 mg compared to placebo (*p* < 0.001). Plasma cGMP levels were significantly elevated following administration, correlating with BP reductions. The prolonged half-life of XXB750 (∼15–25 days) supports its potential for once-monthly subcutaneous dosing, which could improve adherence in patients requiring long-term treatment.

NPR1 agonism offers several advantages over traditional RAAS inhibitors. NPR1 activation induces vascular relaxation through cGMP-mediated pathways, increases sodium and water excretion, and reduces intravascular volume. Unlike ACE inhibitors or ARBs, NPR1 agonists bypass RAAS blockade, reducing the risk of hyperkalemia and other hormonal side effects (79, 82). Additionally, its monthly dosing regimen could improve adherence compared to daily medications.

Despite its promise, several challenges remain for XXB750. XXB750 is still in Phase 2 clinical trials and has not yet been approved for hypertension or HF treatment. As a novel agent requiring subcutaneous administration, XXB750 may be more expensive than oral antihypertensive therapies. Long-term cardiovascular outcomes remain unproven. Lastly, its efficacy may be limited to specific populations such as those with resistant hypertension or HFrEF.

#### 2.1.10 Hypertension vaccines

Several hypertension vaccines targeting the RAAS are currently in clinical trials or have been investigated in earlier phases (83). These vaccines aim to reduce BP by inducing an immune response against key components of the RAAS, such as angiotensin II (84).

**AngQb-Cyt006**, developed by Cytos Biotechnology, is one of the most extensively studied hypertension vaccines (85). It is based on virus-like particles conjugated with angiotensin II peptides to stimulate the production of anti-Ang II antibodies. In a Phase IIa trial involving 72 patients with mild-to-moderate hypertension, participants received subcutaneous injections of either 100 µg, 300 µg, or placebo at weeks 0, 4, and 12. The high-dose group (300 µg) showed significant reductions in daytime ambulatory SBP (−9 mmHg) and DBP (−4 mmHg) at week 14 compared to placebo (*p* < 0.05). Additionally, the vaccine reduced the early morning BP surge by −25 mmHg systolic and −13 mmHg diastolic compared to placebo (85, 86). No serious adverse events were reported, and the vaccine was well-tolerated; however, further development was discontinued due to lower efficacy compared to existing RAAS inhibitors.

**AGMG0201** is a DNA-based angiotensin II vaccine currently undergoing Phase I/IIa trials (87). This vaccine uses a DNA platform to induce long-lasting antibody responses against Ang II. In a recent trial, AGMG0201 was administered to patients with mild-to-moderate hypertension in two doses (low and high). The vaccine induced measurable anti-Ang II antibody titers in most participants, particularly in the high-dose group (88). While safety and tolerability were confirmed—with no severe adverse events reported—its efficacy in reducing BP has not yet been fully evaluated. Advanced clinical studies are expected to investigate its antihypertensive effects further.

ATRQβ-001 is another promising candidate developed using peptide fragments derived from the angiotensin II type 1 receptor (AT1R) conjugated with Qβ bacteriophage virus-like particles. Preclinical studies in spontaneously hypertensive rats (SHRs) demonstrated significant BP reductions of up to −19 mmHg systolic (*p* < 0.01). The vaccine also prevented target organ damage, including cardiac hypertrophy and renal fibrosis. However, ATRQβ-001 has not yet entered human trials (84).

Hypertension vaccines hold promise in reducing the burden of cardiovascular diseases by addressing the root causes of hypertension rather than merely managing its symptoms (84). By providing sustained antibody-mediated inhibition of key hypertensive factors, these vaccines could decrease the incidence of complications such as stroke, myocardial infarction, and CKD.

Compared to daily oral antihypertensive medications, vaccines offer the advantage of long-lasting effects with fewer doses (e.g., biannual injections). This could improve adherence in patients who struggle with daily medication administration regimes. However, current data suggest that vaccines are less effective than existing RAS inhibitors in lowering BP significantly.

However, challenges remain in optimizing vaccine formulations, ensuring long-term efficacy, and addressing potential immunological side effects (89). Ongoing research and future clinical trials are essential to fully establish the role of hypertension vaccines in clinical practice and to determine their place within the broader spectrum of hypertension management strategies.

### 2.2 Endothelin modulators

#### 2.2.1 Dual endothelin receptor antagonists (ET-A and ET-B)

**Aprocitentan** is a dual endothelin receptor antagonist, targeting both endothelin A (ET-A) and endothelin B (ET-B) receptors and effectively inhibiting the strong vasoconstrictive and proliferative actions of endothelin-1, a potent endogenous vasoconstrictor. Compared to earlier endothelin receptor antagonists that were more selective for either ET-A or ET-B receptors, aprocitentan offers an improved safety profile and enhanced efficacy, with a lower rate of the adverse effects such as hepatotoxicity and limited BP reduction observed with previous agents (90).

The PRECISION trial, a phase 3 clinical study, has demonstrated the efficacy of aprocitentan in lowering BP among patients with resistant hypertension. In this trial, 730 participants with resistant hypertension were randomized to receive one of two doses of aprocitentan or a placebo for an initial period of four weeks. It was then followed by a single-blind phase where all participants received a high dose of aprocitentan (25 mg) for 32 weeks. After that period, participants were re-randomized to continue with the high-dose aprocitentan or switch to placebo for an additional 12 weeks (91).

The results revealed that the higher dose of aprocitentan significantly reduced office SBP (the primary endpoint) by an average of 4 mmHg more than placebo. Aprocitentan demonstrated a more pronounced effect on night-time ambulatory BP (7 mmHg reduction) compared to daytime measurements (5 mmHg reduction), a pattern not typically seen with conventional antihypertensive therapies. Additionally, aprocitentan was associated with a substantial decrease in albuminuria by approximately 30%, and it showed the most significant BP-lowering effects in participants with albuminuria and CKD stages 3 and 4. However, the trial also identified that 18% of participants on the higher dose experienced fluid retention, with severe cases leading to discontinuation of the medication in seven participants (90–93).

Aprocitentan was generally well-tolerated across all trial phases, but fluid retention was a notable adverse event, occurring in 9% of patients on the 12.5 mg dose and in 18% on the 25 mg dose during the double-blind phase, compared to only 2% in the placebo group. Severe cases led to treatment discontinuation in seven participants. No treatment-related deaths were reported, and other adverse events were mild to moderate (94).

Aprocitentan was approved by the FDA on March 19, 2024, for use in combination with other antihypertensive medications to lower blood pressure in adult patients whose hypertension is not adequately controlled by other treatments (95). Additionally, it has been approved by the UK's Medicines and Healthcare products Regulatory Agency (MHRA) for similar indications (96).

As for the limitations, fluid retention remains a concern, particularly at higher doses, the cost of it may be more expensive than traditional antihypertensive medications, and the BP reductions are smaller than those achieved with some existing therapies. Moreover, aprocitentan is contraindicated in pregnancy due to potential embryo-fetal toxicity (97).

#### 2.2.2 Endothelin A receptor selective antagonists (ERAs)

A novel endothelin A receptor selective antagonist that shows promise in the treatment of hypertension and associated cardiovascular complications is **zibotentan**, whose mechanism involves selectively blocking the endothelin A receptor. By inhibiting the vasoconstrictive effects of this potent vasoconstrictor peptide - endothelin-1, this ERA leads to vasodilatation and ultimately reduces systemic BP (98). Unlike earlier endothelin receptor antagonists, zibotentan's high selectivity for ET-A minimizes adverse effects related to ET-B receptor blockade, such as fluid retention and nasal congestion.

The PRIZE trial, a randomized, double-blind, placebo-controlled crossover study, investigated zibotentan in 118 patients with microvascular angina. Participants received zibotentan 10 mg daily for 12 weeks. While the primary outcome of treadmill exercise duration did not improve significantly (mean difference: −4.26 s; 95% CI: −19.60–11.06; *p* = 0.59), zibotentan demonstrated significant reductions in BP. DBP decreased by −6.19 mmHg (95% CI: −8.41 to −3.97; *p* < 0.001), and SBP decreased by −5.49 mmHg (95% CI: −8.49 to −2.50; *p* < 0.001). Additionally, zibotentan lowered glycated hemoglobin (HbA1c) and low-density lipoprotein cholesterol (LDL-C), suggesting potential metabolic benefits (95, 99).

The ZENITH-CKD Phase IIb trial (100) evaluated the combination of zibotentan with dapagliflozin (an SGLT2 inhibitor) in patients with CKD and albuminuria already receiving standard care. A total of 447 participants were randomized into three groups: dapagliflozin alone (10 mg daily), zibotentan 0.25 mg/dapagliflozin, or zibotentan 1.5 mg/dapagliflozin for 12 weeks. The primary endpoint was the change in urinary albumin-to-creatinine ratio (UACR). Zibotentan combined with dapagliflozin reduced UACR by an additional 35% compared to dapagliflozin alone (*p* < 0.001). Secondary outcomes included BP reduction, with SBP decreasing by −7 mmHg in the zibotentan/dapagliflozin group compared to placebo (*p* < 0.01). The combination therapy was generally well-tolerated, with fluid retention reported in less than 5% of participants. Long-term cardiovascular and renal benefits require further investigation in Phase III trials.

Compared to traditional antihypertensive agents like ACE inhibitors or ARBs, zibotentan offers unique benefits by targeting the endothelin pathway, which is implicated in resistant hypertension and CKD progression. Its combination with dapagliflozin leverages complementary mechanisms—endothelin inhibition reduces vascular stiffness while SGLT2 inhibition promotes osmotic diuresis—offering enhanced efficacy in reducing albuminuria and BP.

Another ERA, darusentan, has been extensively studied for its potential to treat resistant hypertension (101–105). However, despite promising efficacy in clinical trials, darusentan has not received regulatory approval due to safety concerns and limitations in its clinical development.

In a Phase 2 trial, darusentan reduced SBP by −11.5 mmHg (*p* = 0.015) and DBP by −6.3 mmHg (*p* = 0.002) at the highest dose of 300 mg daily, with significant reductions in 24-h ambulatory BP. Adverse events included mild-to-moderate edema (20%) and headache (15%) (102).

The DORADO Phase 3 trials further evaluated darusentan in resistant hypertension. In the DORADO-311 trial (NCT00389779), darusentan reduced mean SBP by −8.6 mmHg, −16.5 mmHg, and −18.1 mmHg at doses of 50 mg, 100 mg, and 300 mg daily, respectively (*p* < 0.001) (103). Ambulatory BP reductions were also significant, but fluid retention occurred in up to 32% of patients at higher doses (104). Despite its efficacy, darusentan's development was discontinued due to safety concerns related to fluid retention and its modest improvements compared to existing therapies.

The PATHWAY-2 trial established spironolactone as the most effective add-on therapy for resistant hypertension, demonstrating greater BP reductions than other antihypertensive agents such as bisoprolol or doxazosin (105). While darusentan and other ERAs offer an alternative mechanism of action, their efficacy and tolerability have not surpassed that of spironolactone, which remains the benchmark for add-on therapy in resistant hypertension according to current evidence (105). Thus, although darusentan highlighted the potential of endothelin receptor antagonists, safety concerns and lack of superior efficacy have limited its clinical adoption.

### 2.3 Vascular tone modulators

#### 2.3.1 Soluble guanylate cyclase stimulators (sGCs)

Vasodilatation can be targeted by enhancing the nitric oxide (NO)-cyclic guanosine monophosphate (cGMP) signaling pathway. Soluble guanylate cyclase (sGC) stimulators increase the production of cGMP, which promotes the relaxation of vascular smooth muscle and improves endothelial function. This mechanism not only aids in lowering BP but also contributes to favorable cardiovascular remodeling (106).

**Vericiguat**, an oral sGC stimulator approved for HFrEF, has demonstrated modest BP-lowering effects in clinical trials. While not yet approved for hypertension, its dual action on BP and cardiovascular outcomes suggests potential utility in resistant hypertension, particularly in patients with comorbid HF.

The VICTORIA trial evaluated vericiguat in 5,050 HFrEF patients, demonstrating a 10% reduction in cardiovascular death or HF hospitalization (HR: 0.90; *p* = 0.019). Subgroup analyses revealed consistent efficacy across baseline systolic BP (SBP) levels, including those with SBP <110 mmHg (107, 108). Vericiguat reduced SBP by −4 mmHg and DBP by −2 mmHg compared to placebo, with no excessive hypotension-related adverse events even in vulnerable subgroups (108). These findings suggest that vericiguat's BP-lowering effects could be safely harnessed in hypertensive patients, though further studies are needed to confirm its role in this population.

Despite its promise, vericiguat faces limitations for broader use in hypertension management. The magnitude of BP reduction (−4/−2 mmHg) is modest compared to established antihypertensive therapies, and its cost may limit accessibility in low-resource settings. While generally well-tolerated, fluid retention and symptomatic hypotension were reported in some patients, particularly those with baseline SBP <110 mmHg or concurrent use of ARNIs (108). Further studies are needed to evaluate its long-term efficacy and safety in hypertensive populations and to compare its effectiveness against standard therapies.

### 2.4 Neurohormonal modulators

#### 2.4.1 Galanin receptor agonists

Galanin receptors, particularly GALR1 and GALR2, are expressed in key regions of the brain involved in autonomic control, such as the hypothalamus and brainstem, and by selectively activating these receptors, drug therapies may enact sympatholytic effects such as reduced sympathetic outflow, decreased heart rate, lowered vascular resistance (109). Galnon, a synthetic non-peptide agonist of galanin receptors, has been discussed for other conditions, but also on its potential to modulate sympathetic nervous system activity, a key contributor to hypertension (110).

Microinjection of galanin into the rostral ventrolateral medulla (RVLM) has been shown to significantly reduce sympathetic nerve activity (−37.0 ± 7.2% of baseline), mean arterial pressure (MAP; −17.0 ± 3.5 mmHg), and heart rate (−25.0 ± 9.1 beats/min) in urethane-anesthetized rats (111).

Compared to existing antihypertensive therapies such as beta-blockers or calcium channel blockers, Galnon offers a unique mechanism by addressing sympathetic overactivity directly. However, human trials are currently lacking, and further research is needed to evaluate its efficacy and safety in clinical settings.

#### 2.4.2 Glucagon-like peptide-1 receptor agonist (GLP-1 RAs)

A notable mention in this review represents an innovative class of medications that were initially developed for type 2 diabetes management, and also for resistant obesity treatment, two conditions which not only frequently coexist, but they also coexist with the subject of our review and these are glucagon-like peptide-1 receptor agonists (GLP-1 RAs) (112).

GLP-1 RAs, including **liraglutide, semaglutide, dulaglutide,** and **tirzepatide**, act by stimulating GLP-1 receptors, leading to natriuresis, diuresis, inhibition of the RAAS, and improved endothelial function through increased nitric oxide (NO) production (113–117). Additionally, their ability to reduce body weight—a critical factor in resistant hypertension—further enhances their antihypertensive potential.

A few clinical studies have tried to unveil GLP-1 RAs effects on lowering BP. A meta-analysis showed a mean SBP reduction of 2.5 mmHg within the first 2 weeks of treatment (61). Semaglutide and tirzepatide have demonstrated even greater SBP reductions, while tirzepatide showed the most pronounced effect among GLP-1 RAs in the SURPASS trial, being a dual GLP-1 and GIP receptor agonist (116). The LEADER study demonstrated that liraglutide reduces the risk of major cardiovascular events in type 2 diabetes patients with elevated cardiovascular risk, many of whom also had hypertension (117).

High costs and common side effects like nausea and vomiting may limit accessibility and adherence. While trials such as LEADER and SURPASS have demonstrated cardiovascular benefits, long-term studies specifically targeting resistant hypertension are lacking. The high prevalence of obesity (56%–91%) among resistant hypertension patients makes GLP-1 RAs an attractive therapeutic option due to their dual effects on body weight and BP.

### 2.5 Inflammatory and vascular adhesion modulatorss

#### 2.5.1 Vascular adhesion protein-1 (VAP-1) inhibitors

A distinctive pathway targeting BP regulation is specifically targeting vascular adhesion protein-1 (VAP-1) (118). This protein is a molecule involved in inflammatory responses and in endothelial dysfunction, by playing a role in leukocyte traffic and adhesion, leading to chronic inflammation (119).

This class is presently represented by **BTT1023 (Timolumab)**, a novel vascular adhesion protein-1 (VAP-1) inhibitor (120). By inhibiting VAP-1, BTT1023 aims to attenuate the inflammatory response and enhance endothelial function, thereby facilitating vasodilation and reducing vascular resistance.

BTT1023 has been evaluated in Phase I and II clinical trials for inflammatory conditions such as rheumatoid arthritis and primary sclerosing cholangitis (PSC). The Phase II BUTEO trial (121) assessed BTT1023's safety, pharmacokinetics, and efficacy in PSC patients. The study demonstrated that BTT1023 was well-tolerated at doses up to 8 mg/kg, with no cytokine release syndrome or dose-limiting toxicities reported. While the trial primarily focused on liver disease outcomes, the observed anti-inflammatory effects suggest potential benefits for hypertension management driven by chronic inflammation.

While BTT1023 (Timolumab) represents a promising candidate for inflammatory hypertension management due to its novel mechanism targeting VAP-1, dedicated trials evaluating its efficacy in lowering BP are needed to establish its role in hypertension treatment. Future studies should focus on identifying patient subgroups most likely to benefit from this approach while addressing barriers related to cost and accessibility.

## 3 Clinical implications and future directions

While being effective for many people around the globe, traditional antihypertensive therapies still fall short in achieving optimal BP control in a subset of patients, particularly those with resistant hypertension (3, 4).

Novel therapeutic agents for hypertension are being studied and they show promising results in treating hypertension and resistant hypertension in preclinical studies and clinical studies (phase 1 to phase 3), while also some of these new drugs have already been approved to use in hypertension, such as ARNI and nsMRAs (esaxerenone) in few countries.

The introduction of innovative treatments such as AT2R agonists [e.g., Compound 21 (C21)], nsMRAs like finerenone and esaxerenone, mineralocorticoid receptor degraders (MRDs) such as XXB750, selective aldosterone synthase inhibitors (ASAIs - e.g., baxdrostat), aminopeptidase A inhibitors (APAIs - e.g., firibastat), silencing angiotensin agents via RNA (siRNA- e.g., zilebesiran, IONIS-AGT-LRx), emerging hypertension vaccines (e.g., CYT006-AngQb), dual endothelin receptor antagonists (e.g., aprocitentan), sGC stimulators (e.g., vericiguat), neurohormonal modulators as galanin receptor agonists (e.g., galnon) and Glucagon-like peptide-1 receptor agonists (GLP-1 RAs), and last but not least, vascular adhesion protein-1 (VAP-1) inhibitors (e.g., BTT1023 - Timolumab) collectively represent a multifaceted approach to overcoming the limitations of existing therapies.

Despite the promising advancements, several areas necessitate further investigation to fully realize the clinical potential of these novel therapies (Table 1). Ongoing and future clinical trials are essential to establish long-term efficacy, safety, and optimal dosing regimens. Comparative studies evaluating these new agents against established therapies will elucidate their relative benefits and inform clinical guidelines. Additionally, research into combination therapies could explore synergistic effects, offering comprehensive BP control and addressing multiple pathophysiological mechanisms concurrently.

Personalized medicine approaches, leveraging genetic and biomarker profiling, may further enhance the efficacy of these therapies by identifying patient subgroups most likely to benefit from specific treatments. Addressing challenges related to cost, accessibility, and regulatory approvals will also be critical to ensure these advancements translate into widespread clinical practice.

## 4 Conclusions

The integration of these innovative therapeutic agents into hypertension management holds significant promise for improving BP control, particularly in patients with resistant hypertension and various comorbid cardiovascular conditions. By targeting diverse mechanisms underlying hypertension, these treatments may offer enhanced efficacy, improved safety profiles, and greater patient adherence. Continued research and clinical validation will be pivotal in establishing their roles within antihypertensive therapy, ultimately contributing to reduced cardiovascular morbidity and mortality on a global scale.
